# Effects of touch-screen technology usage on the hand skills dataset

**DOI:** 10.1016/j.dib.2020.106358

**Published:** 2020-09-30

**Authors:** Nurul Afiq'ah Aman, Chi-Wen Chien, Jenni Judd, Ahmad Zamir Che Daud

**Affiliations:** a Centre of Occupational Therapy Studies, Faculty of Health Sciences, Universiti Teknologi MARA (UiTM) Selangor, Puncak Alam Campus 42300 Selangor, Malaysia; b Department of Rehabilitation Sciences, Hong Kong Polytechnic University, Hung Hom, Kowloon, Hong Kong (SAR), China; c School of Health, Medical and Applied Science, Centre for Indigenous Health Equity Research, Central Queensland University, Bundaberg, Australia

**Keywords:** Child behaviour, Child development, Hand, Motor skills, Screen time

## Abstract

This data article describes the hand skills of pre-school children between five and six years old from five schools under the Ministry of Education Malaysia. These data may be used in a journal article later to show the effects of touch-screen technology usage on hand skills of pre-school children. Demographic characteristics, hand skills ability and frequency of touch-screen technology usage data that was collected from August to September 2019. These data may be used in a future systematic review, meta-analysis and meta-regression analysis to conclude the effects of touch-screen technology usage on children's hand skills. Parents, teachers and health practitioners may refer to these data to note the effects of touch-screen technology usage on hand skills of pre-school children.

## Specifications Table

**Table:** 

Subject	Nursing and Health Profession: Occupational Therapy
Specific subject area	Touch screen technology usage and its' effects on hand skills for pre-school children
Type of data	Fig.s and tables
How data were acquired	Demographic information questionnaire, Assessment of Children's Hand Skills (ACHS) and Children's Hand Skills ability Questionnaire (CHSQ). Demographic information questionnaire is provided as a supplementary file.
Data format	Data are in raw format and have been analysed. Data files are uploaded along with this article.
Parameters for data collection	Participants are typically developing pre-school children aged between five and six years old, attending the Ministry of Education (MOE) pre-schools and engaging in touch-screen technology. Children were divided into two groups; high usage touch-screen technology (HUTSTG) and, low usage touch-screen technology (LUTSTG) group. The children who engaged in touch-screen technology (either phone or tablet) for less than two hours per day were allocated in LUTSTG, and those engaged for more than two hours per day were allocated in the HUTSTG. The children diagnosed with diseases or disorders associated with developmental delays were excluded from the data collection process.
Description of data collection	Parents of pre-school children completed the demographics information questionnaire about their children and touch-screen technology usage. Parents were asked to complete the CHSQ to evaluate the uses of the hands by the children age 2 – 12 years old while performing activities of the daily life from the perspectives of the parents or the caregivers [1]. An occupational therapist then conducted a performance-based assessment with all participants using the ACHS. This performance-based assessment was used to assess the actual performance of the child's hand skills in a real-life context when engaging in several types of daily activities in everyday settings [2]. Hand skills were evaluated while the children performed two or three selected activities out of the 22 activities in the ACHS for 20 – 30 minutes at their pre-schools. Selection of activities was informed by the parents' responses from the CHSQ.
Data source location	Ministry of Education (MOE) pre-schools, Johor, Malaysia
Data accessibility	All data and supplementary files are included with this article.

## Value of the Data

• The data provided in this article reveals the effects of touch-screen technology on children's hand skills.
• The data benefits parents, teachers and health practitioners to take note of the effects of touch-screen technology usage on children's hand skills.
• These data may be used in a future systematic review, meta-analysis and meta-regression analysis to conclude the effects of touch-screen technology usage on children's hand skills.
• These data will enable researchers and practitioners to find the trends on the effects of touch-screen technology to children such as hand skills, cognitive, visual perception, interaction and others.

## 1 Data Description

**Fig. 1:**
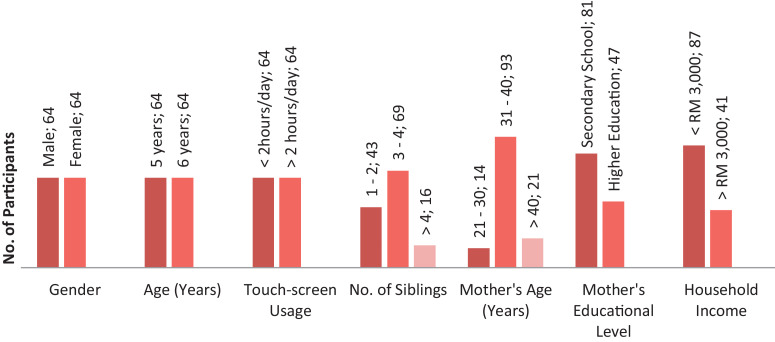
Participant's demographic characteristics.

The power analysis for this dataset was considered according to the results from a comparable study [3]. A total of 128 participants with 64 pre-school children in each group were recruited to achieve the power of 0.80 to detect the medium-effect size (*d* = 0.50) and *α* = 0.05. Fig. 1 shows the data of the participant's demographic characteristics. Participants were recruited from five Ministry of Education (MOE) pre-schools in Johor, Malaysia.

**Fig. 2.1:**
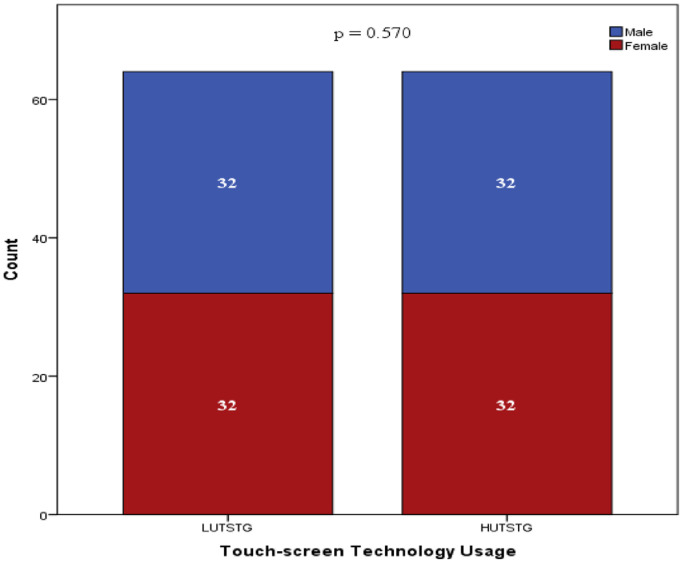
Gender and touch-screen technology usage.

**Fig. 2.2:**
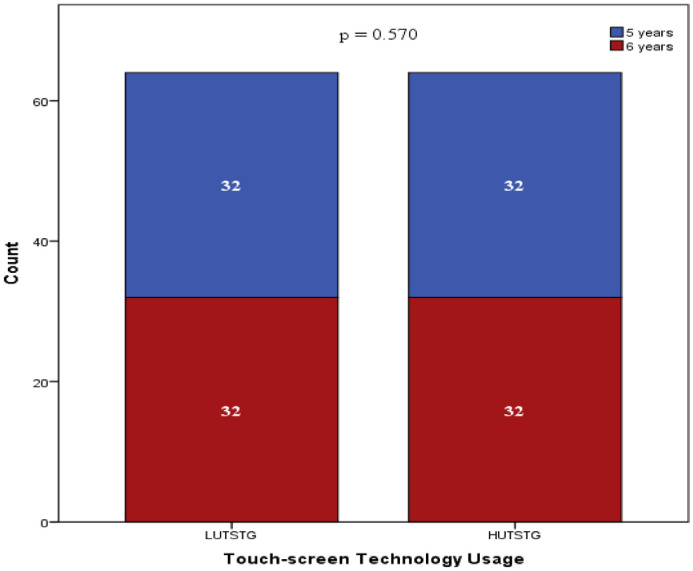
Age and touch-screen technology usage.

**Fig. 2.3:**
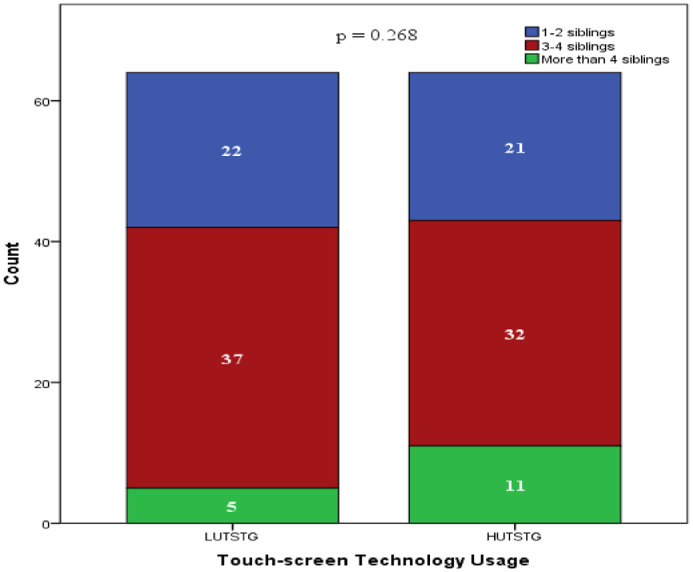
No. of siblings and touch-screen technology usage.

Both HUTSTG and LUTSTG groups consisted of 64 pre-school children; the number of genders (male and female) and age (five years and six years) was also balanced between the groups (as shown in Fig. 2.1 and Fig. 2.2). However, other demographic characteristics were not controlled. Fig. 2.3–2.6 show the data on the number of siblings, mother's age, mother's educational level and household income for HUTSTG and LUTSTG group. Raw data as in the Supplementary Data 1.

**Fig. 2.4:**
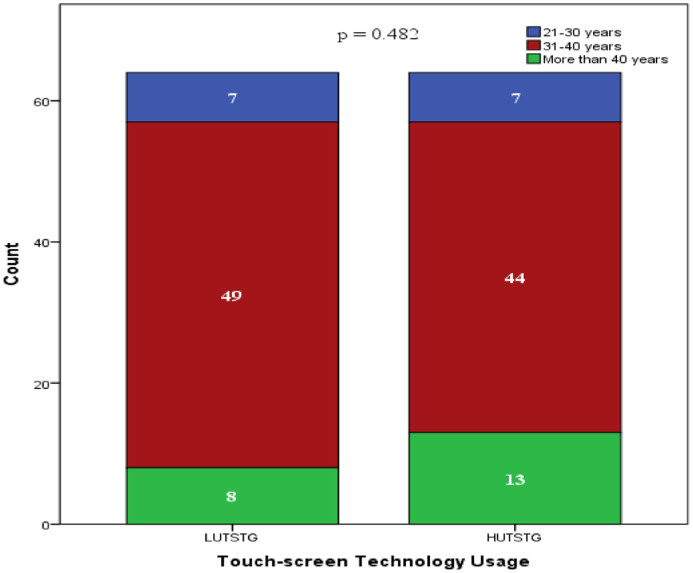
Mother's age and touch-screen technology usage.

**Fig. 2.5:**
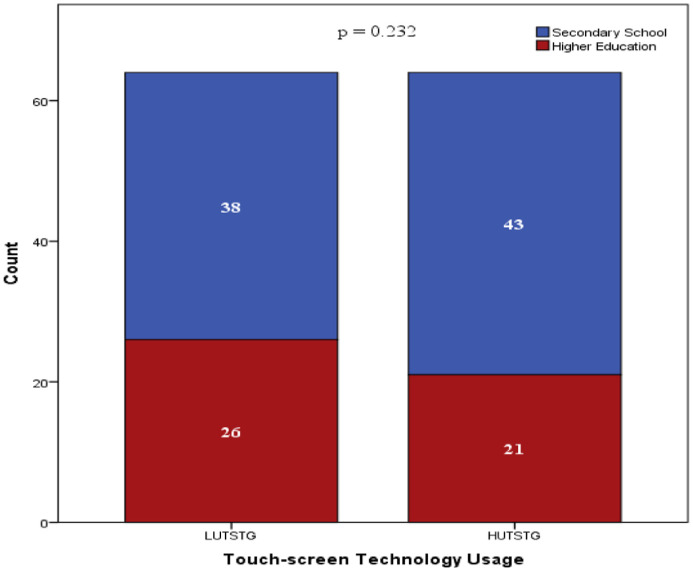
Mother's educational level and touch-screen usage.

**Fig. 2.6:**
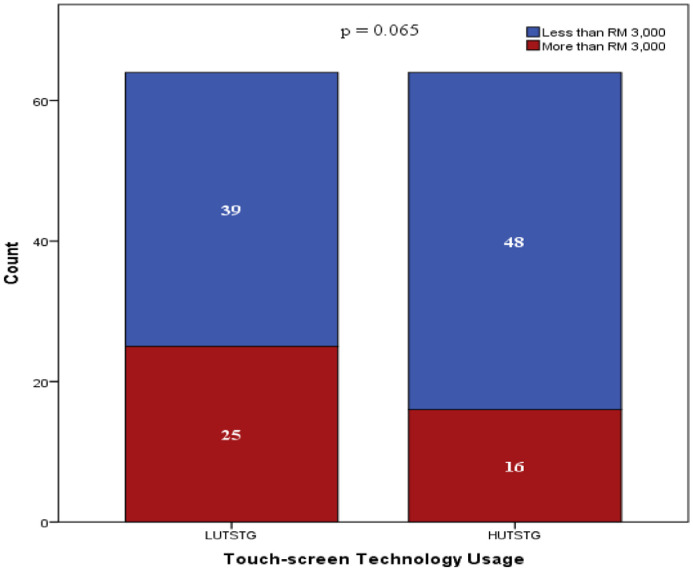
Household Income and touch-screen technology usage.

An independent T-test was used to compare the hand skills of children in the LUTSTG and HUTSTG groups based on average scores of CHSQ and composite scores of ACHS. Table 1 shows the effects of touch-screen technology usage on hand skills. Raw data for analysis results as in the Supplementary Data 1.

**Table 1:** Touch-screen technology usage and hand skills.

		M ± SD			
Hand skills		LUTSTG	HUTSTG	Effect size	*P*-value
Performance-based assessment (ACHS)		2.702 ± 1.808	0.956 ± 1.266	1.119	< 0.001
Parents’ reported questionnaire (CHSQ)	Play/leisure domain	2.908 ± 0.176	2.788 ± 0.199	0.639	< 0.001
	School/education domain	2.929 ± 0.170	2.784 ± 0.206	0.768	< 0.001
	ADL domain	2.990 ± 0.041	2.945 ± 0.100	0.589	0.001

## 2 Experimental Design, Materials and Methods

Consent was obtained, and data collection procedures were explained to the parents. Demographic information questionnaire was completed by parents that comprised of three parts which are; (1) Demographic information of children (gender (male and female), age, date of birth and number of siblings (stratified to 1-2, 3-4 and more than 4 siblings)); (2) Family background (mother's age (21 – 30, 31 – 40 and more than 40 years), mother's educational level (primary school, secondary school and higher education) and household income (less than RM 3,000 and more than RM 3,000)); and (3) Touch-screen technology usage (accessed to touch-screen technology (yes and no) and average used of touch-screen technology (less than two hours per day and more than two hours per day)). Table 2 shows the components of questionnaires and measurement types used in data analysis. Demographic information questionnaire as shown in the Supplementary File 1.

**Table 2:** Components of questionnaire and measurement type.

Part	Questions	Measurement type
A (Demographic Information)	Children's gender	Nominal (Categories: 1= Male; 2= Female)
	Children's age	Ordinal (Categories: 1= 5 years; 2= 6 years)
	Date of birth	-
	Number of siblings	Ordinal (Categories: 1= 1-2 siblings; 2= 3-4 siblings; 3= More than 4 siblings)
B (Family Background)	Mother's age	Ordinal (Categories: 1= 21-30 years; 2= 31-40 years; 3= More than 40 years)
	Mother's educational level	Ordinal (Categories: 1= Secondary school; 2= Higher education)
	Household income	Ordinal (Categories: 1= Less than RM 3,000; 2= More than RM 3,000)
C (Touch-screen Technology Usage)	Access to touch-screen technology	Nominal (Yes or No)
	Average of touch-screen usage per day	Nominal (Categories: 1= Less than 2 hours per day; 2= More than 2 hours per day)

The CHSQ was used to evaluated the use of the children's hands while performing daily life activities from the perspective of parents or the caregivers' [4]. This assessment comprised of 22 hand skills activities that are divided into three domains of leisure and play (8 activities), school/education (6 activities), and activities of daily living (6 activities). Likert scales with three levels were used in CHSQ that are; 1 (extremely difficult), 2 (difficult), and 3 (no difficulty) indicating the difficulty level while performing the activities. The "not applicable" will be marked when the child has not attempted to perform the activity in the last three months. This assessment can be used as a companion assessment to know the baseline of the range of the disabilities before evaluating using the performance-based assessment of ACHS. Average scores of CHSQ were calculated for each domain and used in the data analysis. Raw scores and the calculated average for CHSQ assessment is in the Supplementary Data 2.

ACHS was used to assess the actual performance of the children's hand skills when involved in several types of daily activities in everyday settings [2]. This ACHS comprises of 5 categories of hand skills items; (1) hand skills without interacting with objects (manual gesture, body contact); (2) arm-hand use (reaching, turning, carrying, throwing, catching, moving, stabilising); (3) adaptive skilled hand use (grasping, holding, in-hand manipulating, releasing, isolated finger movements); (4) bimanual use object-related hand skills (transferring, using both hands simultaneously, using both hands cooperatively); and (5) general quality of hand skills (accuracy, pace, movement quality). ACHS is rated on a 6-point rating scale. A score of 6 indicates very effective hand skill performance, whereas a score of 1 indicates very ineffective hand skill performance. Three scoring methods are used for this assessment; (1) Item raw scores; (2) Activity percentage scores; and (3) Child composite scores. For this dataset, child composite scores were used and were computed by the developer of the assessment. Supplementary Data 3 shows the raw scores for each item of ACHS and Supplementary Data 4 shows the computed composite scores of ACHS.

## Ethics Statement

The Ethics Review Committee of Universiti Teknologi MARA (UiTM) (REC/223/19) approved the study. Additionally, permissions from the Ministry of Education (KPM.600-3/2/3-eras-3447) and Johor State Education Department (JPNJ.PP.600-1/1/2Jld.2-42) were gained as the data collection process involved the pre-school children under the Ministry of Education, Malaysia. The procedures were explained to the parents, and their written consent was obtained before the pre-school children and their parents.

## Declaration of Competing Interest

The authors declare that they have no known competing financial interests or personal relationships which have, or could be perceived to have, influenced the work reported in this article.

## Appendix Supplementary Materials

Image, application 1

Image, application 2

Image, application 3

Image, application 4

Image, application 5
