# Supplementary material for: Effects of touch-screen technology usage on the hand skills dataset
Source: Data Brief. 2020 Sep 30;33:106358. doi: 10.1016/j.dib.2020.106358 (PMC7554022; doi:10.1016/j.dib.2020.106358)
Supplement: Supplementary file 5 [file mmc5.pdf]

ID :

PART A : DEMOGRAPHIC INFORMATION OF THE CHILDREN

Instruction :
This part consists of questions regarding your child's background. Kindly fill in the answer and tick (/) the most appropriate box respectively. All questions must be answered.

1) **Gender** :

   Male ☐

   Female ☐

2) **Age** : ____________ years

3) **Date of birth ** : ____________

4) **Number of siblings** :

   1 – 2 ☐

   3 – 4 ☐

   More than 4 ☐

ID :

PART B : FAMILY BACKGROUND

Instruction :
This part consists of questions regarding your family background. Kindly tick (/) the most appropriate box respectively. All questions must be answered.

1) **Mother's Age :**

21 – 30 years ☐

31 – 40 years ☐

More than 40 years ☐

2) **Mother's Educational Level :**

Primary School ☐

Secondary School ☐

Higher Education ☐

3) **Household income :**

Less than RM 3,000 ☐

More than RM 3,000 ☐

ID :

PART C : TOUCH-SCREEN TECHNOLOGY USAGE

Instruction :
This part consists of questions regarding your child's touch-screen technology usage. Kindly tick (/) the most appropriate box respectively. All questions must be answered.

1) Did your child have accessed to the touch-screen technology?

Yes ☐

No ☐

2) How often in average per day does your child engaged in the touch-screen technology?

Less than 2 hours per day ☐

More than 2 hours per day ☐
